# The Antioxidant Effect of Curcumin on Cochlear Fibroblasts in Rat Models of Diabetes Mellitus

**Published:** 2017-07

**Authors:** Tengku-Siti-Hajar Haryuna, Delfitri Munir, Ana Maria, Jenny Bashiruddin

**Affiliations:** 1 *Department of Otorhinolaryngology-Head and Neck Surgery, Faculty of Medicine, Universitas Sumatera Utara, Medan 20155, Indonesia*; 2 *Department of Otorhinolaryngology-Head and Neck Surgery, Faculty of Medicine, Universitas Indonesia, Jakarta 10430, Indonesia*

**Keywords:** Antioxidants, Curcumin, Cochlea, Diabetes mellitus, Oxidative stress, Superoxide dismutase

## Abstract

**Introduction:**

The aim of this study was to investigate the potential of curcumin as an antioxidant to increase the expression of superoxide dismutase (SOD) in fibroblasts of the cochlear lateral wall in rat models of diabetes mellitus.

**Materials and Methods:**

Twenty-four male Wistar rats Rattus norvegicus were randomly divided into six groups: group 1 as the control group; group 2 as the diabetic group; group 3 and 4 as the diabetic groups that received curcumin therapy of 200 and 400 mg/kg b.w. for 3 days, respectively; and group 5 and 6 as the diabetic groups that received curcumin therapy of 200 and 400 mg/kg b.w. for 8 days, respectively. All rats underwent termination and necropsy procedure on their temporal bones for immunohistochemical assay to determine the expression of SOD.

**Results:**

The decreased expression of SOD was detected in the diabetic group (without curcumin treatment). The treatment of curcumin at doses of 200 and 400 mg/kg b.w. for 3 and 8 days led to significant differences (P<0.05) in the expression of the SOD compared to diabetic group (without curcumin treatment). No significant differences were found in terms of dose and duration of curcumin administration on the expression of SOD.

**Conclusion:**

Curcumin may act as an antioxidant against oxidative stress due to diabetes mellitus via increased expression of SOD on cochlear fibroblasts in rat models of diabetes mellitus.

## Introduction

Diabetes mellitus is a chronic metabolic disease affecting about 8.5% of the world population (1). Diabetes is characterized by high blood sugar levels due to absolute and relative insulin deficiency. Diabetes has the potential to cause severe morbidity due to the complications to various organs of the body, associated with microvascular damage and neuropathy affecting the retina, renal, peripheral arteries, and peripheral nerves (1,2).

Pathological changes in diabetes mellitus can injure blood vessels and the nervous system of the inner ear, leading to hearing loss. Some of the mechanisms are related to cochlear microangiopathy, perilymphatic hyperglycemia, auditory neuropathy, and diabetic encephalo- pathy (2,3).

Chronic hyperglycemia in diabetes mellitus results in a buildup of free radicals, causing damage to lipids, proteins, and nucleic acids. Elevated levels of free radicals in diabetes mellitus are associated with non-enzymatic glycation of proteins, autoxidation of glucose, activation of the polyol pathway, increased expression of advanced glycation end products (AGEs)-receptor, overactivity of hexosamine pathway, and activation of various Protein Kinase C (PKC) isoforms (4-6).

Oxygen free radicals are molecules containing one or more unpaired electrons in their outermost shells, formed in aerobic cellular metabolism. Free radicals are highly reactive and can bind to various molecules that ultimately cause damage to nucleic acids, proteins, and lipid membranes. Under normal circumstances, free radicals will be defeated by the antioxidant defense system. Various natural antioxidants are available to scavenge free radicals and prevent oxidative damage. Natural antioxidants can exist in enzymatic antioxidants such as catalase, superoxide dismutase (SOD), and glutathione peroxidase. In addition, there are food-derived antioxidants such as vitamin A, C, E, carotenoids, and curcumin (4). 

Curcumin is a bioactive component contained in the cooking spice *Curcuma longa*. Curcumin can modulate the biological activity of various signaling molecules. Curcumin has a variety of biological activities that has been demonstrated in many studies, including: natural antioxidant, anti-inflammatory, antimutagenic, antimicrobial, and anticancer (7-9). A study on the role of curcumin as an antioxidant by Aziza found that curcumin increased the activity of SOD and protected enzymatic antioxidants from the effects of denaturation (10).

This study was conducted to investigate the potential of curcumin as an antioxidant in increasing the expression of SOD in fibroblasts of the cochlear lateral wall in rat models of diabetes mellitus, which can later become the basis for further clinical purposes.

## Materials and Methods

*1. Experimental treatments in animal models*

This experimental study with randomized post-test only control group design was conducted on male *Wistar* rats *Rattus norvegicus*, which were 2-3 months of age and weighed 200-250 mg. All experimental procedures were approved by the Health Research Ethical Committee of North Sumatera c/o Medical School, Universitas Sumatera Utara, Indonesia (project no. 433/ KOMET/FKUSU/2015).

Twenty-four rats were randomized and divided into 6 groups (n= 4). 

Group1: Control group, intraperitoneally injected with citrate buffer on day 1, 0.5% Carboxy Methyl Cellulose (CMC) on day 3 - 5 orally, terminated on day 5. 

Group2: intraperitoneally injected with streptozotocin (60 mg/kg b.w.) on day 1, terminated on day 5. 

Group3: intraperitoneally injected with streptozotocin (60 mg/kg b.w.) on day 1, orally administered with curcumin (200 mg/kg b.w./ day) on day 3-5, terminated on day 5. 

Group4: intraperitoneally injected with streptozotocin (60 mg/kg b.w.) on day 1, orally administered with curcumin (400 mg/kg b.w./ day) on day 3-5, terminated on day 5. 

Group5: intraperitoneally injected with streptozotocin (60 mg/kg b.w.) on day 1, orally administered with curcumin (200 mg/kg b.w./ day) on day 3-10, terminated on day 10. 

Group6: intraperitoneally injected with streptozotocin (60 mg/kg b.w.) on day 1, orally administered with curcumin (400 mg/kg b.w./ day) on day 3-10, terminated on day 10. 

Diabetic groups (group 2- 6) were induced by streptozotocin (Streptozotocin, Bioworld, USA). Diabetes is diagnosed when the glucose concentration is more than 200 mg/dL (11) and verified within 48 hours following the streptozotocin injection (12). Rats which did not meet the criteria were excluded from this study. The examination of glucose concentration was conducted from the tail vein blood sample collection using blood glucose test strips (Gluko DR® Bio Sensor – Allmedicus). The control group (group 1) was treated with citrate buffer and CMC. Diabetic groups were treated with curcumin suspended in 0.5% CMC. CMC is formulated by suspending 0.5 gram CMC in 100 cc aquadest. Curcumin used in this study was derived from Curcuma longa Linnaeus (turmeric) with curcumin content levels of (16.62 ± 0.14)% w/w compared with Standard (identified by thin-layer chromatography and densitometry). After being suspended, curcumin was directly administered into the stomach of each rat via a nasogastric tube.

Rats underwent termination by ether inhalation and necropsy procedure on their temporal bones. Tissue samples were collected and fixated in 10% buffered formalin solutions, then decalcified in EDTA for 4 weeks. All samples underwent a fixation procedure in buffered formaldehyde, followed by dehydration in graded alcohol solutions. Thereafter, they were embedded in paraffin blocks, serially cut into 4 μm thick sections, and put on glass slides.

*2. Immunohistochemical assay*

All samples were immunohistochemically examined for the expression of SOD in cochlear fibroblasts using a primary antibody [polyclonal anti-SOD1 antibodies (Boster Biological Technology Co., Ltd. Cat#: 1345)]. The expression of SOD was investigated using Olympus XC 10 microscope under 40x magnification, marked by brown-stained cytoplasms.

*3. Statistical Analysis*

The data was analyzed with One-Way ANOVA using IBM SPSS Statistics with a significance level of 0.05.

## Results

**Fig 1:**
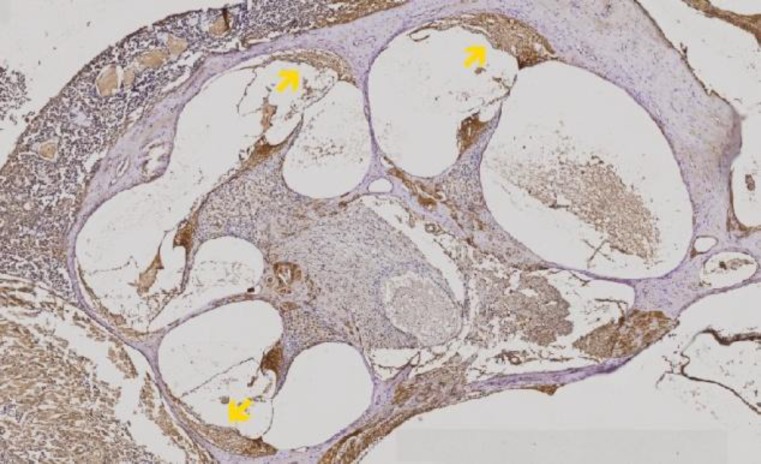
The cochlear lateral wall and supporting tissues with immunohistochemical assay [(marked by yellow arrow) (under 40x magnification

**Fig.2:**
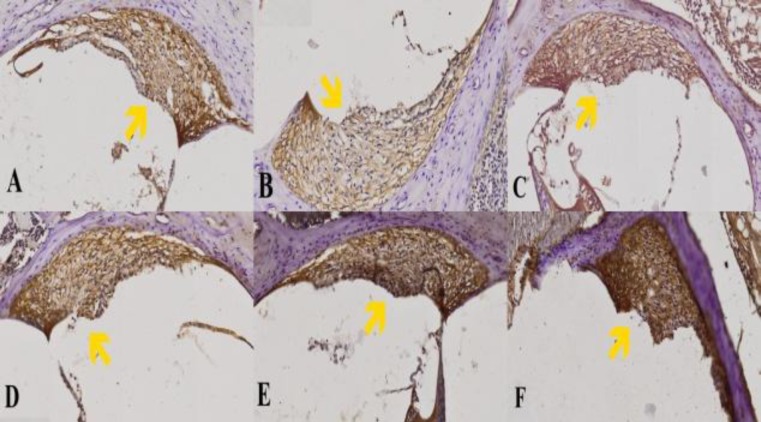
The expression of SOD in each group (under 100x magnification): (A) group 1/control; (B) group 2; (C) group 3; (D) group 4. (E) group 5; (F) group 6. The yellow arrow indicates the expression of SOD in cochlear fibroblasts marked by brown stains

The immunohistochemical assay of rat cochlea was performed and investigated under the microscope to get a detailed view of the cochlear tissue (Fig.1). In group 2 (diabetic group without curcumin treatment), lower density and less SOD-expressed fibroblasts were detected (Fig. 2(B)) compared control group (Fig.2(A)). Curcumin-treated groups (group 3,4,5, and 6) showed higher density and more SOD-expressed fibroblasts (Fig.2 (C, D, E, and F)).

**Chart 1:**
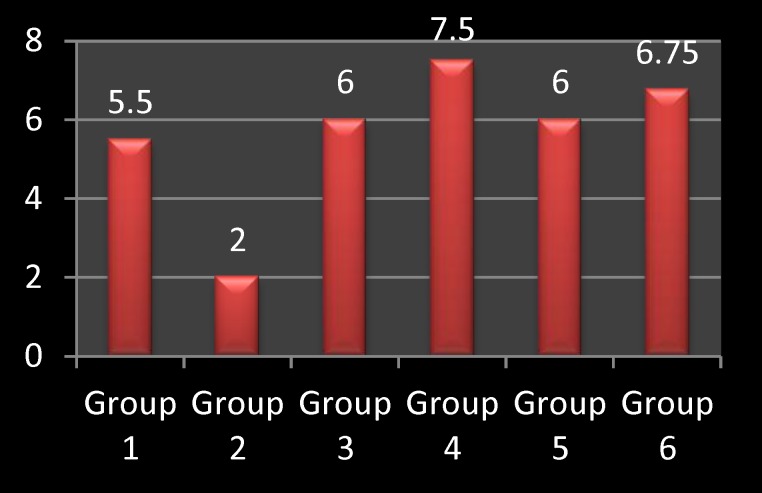
The mean values of SOD expression in each group

The expression of SOD was found to be increased in all diabetic groups treated with curcumin at doses of 200 and 400 mg/kg b.w./day for 5 and 10 days compared to the diabetic group without curcumin treatment and control group, as shown in Chart 1.

**Table 1:** ANOVA test results for the expression of SOD

**Group**		**Mean ± SD**	**P value**
Group 1	Group 2	3.500 ± .759	.003^*^
	Group 3	-.500 ± .759	1.000
	Group 4	-2.000 ± .759	.253
	Group 5	-.500 ± .759	1.000
	Group 6	-1.250 ± .759	1.000
Group 2	Group 3	-4.000 ± .759	.001^*^
	Group 4	-5.500 ± .759	.000^*^
	Group 5	-4.000 ± .759	.001^*^
	Group 6	-4.750 ± .759	.000^*^
Group 3	Group 4	-1.500 ± .759	.956
	Group 5	.000 ± .759	1.000
	Group 6	-.750 ± .759	1.000
Group 4	Group 5	1.500 ± .759	.956
	Group 6	.750 ± .759	1.000
Group 5	Group 6	-.750 ± .759	1.000

Data, in Table 1 above, showed significant differences for the expression of SOD (P<0.05) in all diabetic groups treated with curcumin (group 3,4,5, and 6) compared to the diabetic group without curcumin treatment (group 2). The 80 mg/day dose of curcumin showed statistically insignificant differences compared to the 40 mg/day dose of curcumin regarding the expression of SOD (P> 0.05). A longer duration of curcumin treatment (10 days) also showed statistically insignificant differences compared to the shorter duration of curcumin treatment (5 days) (P> 0.05).

## Discussion

The aim of this study was to analyze the antioxidant properties of curcumin influencing the expression of SOD as the endogenous antioxidant on cochlear fibroblasts in rat models of diabetes mellitus. This study revealed that, in the diabetic samples without curcumin treatment, the expression of SOD was found to be decreased, which is associated with oxidative stress due to hyperglycemia. SOD is a first-line antioxidant enzyme that plays a role in the catalytic process of superoxide radicals into hydrogen peroxide and oxygen. Extracellular SOD is also the only antioxidant enzyme that can act as a scavenger of superoxide in the extracellular compartment (13). In diabetes, the hyperglycemia state inflicts oxidative stress via several pathways. The most important mechanism is the overproduction of superoxide anions through the electron transport chain in mitochondria. The formation of a physiological O2 species (especially superoxide radicals) occurs in the electron transfer by the cytochrome in electron transport chain processes. Hyperglycemia causes the increased production of an electron donor (NADH and FADH2) in the tricarboxylic cycle (14).

The cochlea, especially stria vascular, is a microvascular-dependent organ. The increase in endothelial permeability results in changes in the electrolyte balance within the endolymph, which then influences the process of signal transduction and transmission in the hair cells (6). The organ of Corti, the hearing organ, has a complex structure and potentially becomes a target organ damaged by hyperglycemia. Damage to any part involved in the hearing process will cause hearing loss (15).

Research by Kasznicki et al found a significant decrease in SOD levels in the plasma of patients with diabetes suffering from distal symmetric polyneuropathy (DSPN) (16). Similarly, Palma et al also observed a decreased activity of SOD in the plasma and liver of rat models of diabetes mellitus (17).

This study used curcumin at a dose of 200 mg/kg b.w./day by referring to previous studies (18,19) which determined that dose of curcumin to be able to act as an antioxidant. Furthermore, this study used two different doses and duration of curcumin administration in each group to determine the best dose and duration of curcumin administration. According to previous studies, curcumin has time- and dose-dependent properties, because the duration of administration and the amount of dose used affects the expression of certain genes (20).

This study found that curcumin is able to increase the expression of SOD in diabetic groups. The mechanism of curcumin activity as an antioxidant is based on its radical-scavenging properties against superoxide and hydroxyl oxide, as well as through the upregulation of endogenous antioxidant enzyme expression such as SOD, GSH, and GSHPx (21,22). The upregulation occurs through the mechanism of Nrf2 [(Nuclear factor-(erythroid derived 2) -related factor-2)] gene induction. Nrf2 is located in the cytoplasm and will translocate into the nucleus to initiate the antioxidant pathway (21,23). In addition, curcumin acts through the inhibition of cellular activity producing ROS via the inhibition of the enzyme activity of NADPH oxidase, lipoxygenase/cyclooxygenase, xanthine dehydrogenase, and nitric oxide synthase, consequently improving the bioavailability of cellular antioxidant enzymes (24).

The results obtained in this study are consistent with the study conducted by Jagetia & Rajanikant who found that the administration of curcumin before and after irradiation in mice could increase the expression of SOD (21). Tapia et al also discovered a similar result in an experiment investigating the effects of curcumin toward the oxidant-antioxidant status of kidneys in rats with oxidative stress due to nephrectomy (23). The role of curcumin and the regulation of SOD expression were also found by Jena et al (25), showing the effectiveness of curcumin in improving and increasing SOD expression in the cerebral and cerebellar cortex in rats experiencing oxidative stress due to PTU (6-propyl-2thiouracil).

This study found statistically insignificant differences (P>0.05) in terms of difference doses and durations of curcumin administration, as seen in the groups that received curcumin at doses of 200 mg/kg b.w./day and 400 mg/kg b.w./day for 5 and 10 days, respectively. These results differ from the results of a study conducted by Haryuna et al who found that there is a dose-response relationship between curcumin treatment and SOD expression (26), as well as CAT levels in fibroblasts of cochlear lateral wall in noise-induced rats. This might happen as a result of minimum variation in the dosage of curcumin between groups (200 mg/kg b.w./day and 400 mg/kg b.w./day), thus no difference is observed in terms of SOD expression. Another factor that can influence these results is the nature of curcumin properties, which may act as an antioxidant in small doses but as a prooxidant in large doses (22). Several studies have shown that small doses of curcumin reduce ROS production. However, in large doses, curcumin increases the production of ROS in the study of human leukemia cells (27). Other studies have shown that low doses of curcumin (<10 microM) can prevent the depletion of the antioxidant enzyme GSH, but higher doses cause a gradual decline in GSH of red blood cells experiencing oxidative damage (28).

## Conclusion

According to the results of this study, we concluded that curcumin may act as an antioxidant against oxidative stress due to hyperglycemia via the increased expression of SOD on cochlear fibroblasts in rat models of diabetes mellitus. However, the use of different doses and durations of curcumin administration were found to be statistically insignificant.
